# Development and Evaluation of a Nomogram for INCS Insensitivity in Chinese Adults with Allergic Rhinitis

**DOI:** 10.1155/2023/3027092

**Published:** 2023-04-18

**Authors:** Deping Sun, Lan Liu, Yuqing Yan

**Affiliations:** Department of Otorhinolaryngology Head and Neck Surgery, The Fourth Clinical College of Chongqing Medical University, Chongqing, China

## Abstract

**Objective:**

The objective of this study was to design and validate a nomogram of intranasal corticosteroid (INCS) insensitivity for adult patients with allergic rhinitis (AR).

**Methods:**

Training and validation datasets comprised randomly divided groups of AR patients diagnosed between 2019 and 2022, with a 7 : 3 ratio. These patients were categorized according to their INCS insensitivity status, and LASSO and multivariate logistic regression analyses were conducted to identify associated risk factors. These factors were incorporated into a nomogram for predicting INCS insensitivity. The performance of the nomogram was assessed using receiver operating characteristic (ROC) curves, calibration curves, and discrimination techniques.

**Results:**

In this study, 313 patients were included, of which 120 (38.3%) showed INCS insensitivity. The type of AR, comorbidities, family history of AR, and duration of AR were identified as predictors and incorporated into the nomogram using least absolute shrinkage and selection operator and multivariate logistic regression. The calibration curves showed excellent agreement between predicted and actual probabilities of INCS insensitivity in both the training and validation sets. The area under the curve values observed in the validation set were 0.918 (95% confidence interval, 0.859–0.943), and 0.932 (95% confidence interval, 0.849–0.953) in the training set, indicating strong performance on both sets. Decision curve analysis showed that the constructed nomogram yielded a net clinical benefit for AR patients.

**Conclusion:**

The nomogram constructed from risk predictors of INCS insensitivity in patients with AR demonstrated strong predictive power and enabled clinicians to identify high-risk patients, aiding them in developing an optimal treatment plan for AR.

## 1. Introduction

Allergic rhinitis (AR) is a type I allergic reaction in which the nasal mucosa becomes inflamed due to an IgE-mediated hypersensitive reaction to certain allergens [1]. The estimated global prevalence of AR is between 10 to 40% [2, 3], while in China, it ranges from 11.1%–17.6% from 2005 to 2011 [4, 5]. Typical symptoms of AR that are reported frequently include sneezing, itching, rhinorrhea, obstruction of the nasal cavity, wheezing, redness of the skin, and eye drainage [3]. AR can adversely affect an individual's quality of life due to disturbed sleep and reduced activity and has a significant economic cost [6–9].

Intranasal corticosteroids (INCS) are commonly used to treat moderate-to-severe AR, but their effectiveness varies between individuals; however, even with treatment, a patient may not show a significant improvement in their clinical symptoms [10, 11]. INCS insensitivity is a recognized phenomenon, with potential side effects such as nosebleeds, irritation (including dryness, burning, or tingling), headaches, and even perforation of the nasal septum [12]. Although there are numerous studies on glucocorticoid-insensitivity in asthma [13, 14] and nephrotic syndrome [15], data on this topic in relation to AR is limited. By pinpointing the factors that may lead to INCS insensitivity, it is possible to reduce the unnecessary use of INCS, thereby decreasing the chances of any related complications and allowing for the swift application of more reliable alternatives.

Through utilizing demographic and clinical data such as age, gender, length of history of AR, classification of AR, family history of AR, and comorbid conditions, etc., linked to AR, this study created a predictive model to aid medical professionals in determining INCS insensitivity in AR patients.

## 2. Methods

### 2.1. Participants

This study focused on Chinese individuals aged 18 to 75 years old who had moderate-to-severe AR symptoms that significantly affected their sleep, daily activities, work, or studies [2] and had been receiving INCS mono-therapy for six weeks. Budesonide aqueous suspension (280 *μ*g taken daily) [2, 16, 17] was administered as treatment, and their progress was monitored through follow-up visits.

This study excluded pregnant women, breastfeeding women, individuals who had malignant neoplasms, autoimmune diseases, hepatic or renal diseases, those who had used decongestants in the previous week, those who had taken INCS in the past four weeks, those who had been given systemic corticosteroids in the last eight weeks, those who had been prescribed antileukotrienes or H1 antihistamines in the previous two weeks, those who were undergoing allergen immunotherapy, those who had undergone nasal or sinus surgery, and those who had taken traditional Chinese medicine or acupuncture in the previous two weeks.

AR is characterized by recurrent sneezing, runny nose, nasal itching, nasal congestion, and watering eyes, accompanied by physical signs such as paleness, edema, and increased secretions. This diagnosis is confirmed by positive results from skin prick tests (SPT) and/or the detection of elevated serum-specific IgE levels for allergens [2, 18].

### 2.2. Study Design

**Figure 1:**
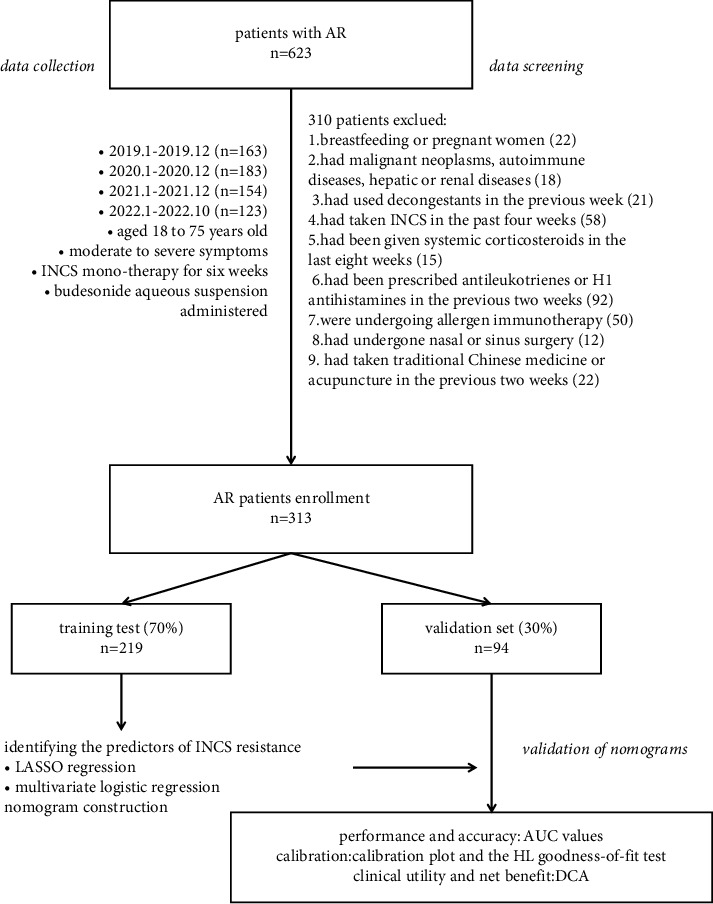
The patient screening process and the study design. AR: allergic rhinitis, INCS: intranasal corticosteroids, LASSO: least absolute shrinkage and selection operator, AUC: curves and areas under the curves, HL: Hosmer–Lemeshow, and DCA: decision curve analysis.

This study aims to find predictive factors for insensitivity to INCS within a training dataset, which is used to develop a nomogram. The performance of this nomogram is evaluated by analyzing the data from the training and validation datasets. An overview of the patient screening process and study design is provided in Figure 1.

### 2.3. Outcomes

Total nasal symptom scores (TNSS) were recorded before and after the patients used INCS. This survey is used to measure the severity of the main AR symptoms and comprises three questions assessing nasal blockage, itchiness or sneezing, and secretions or a runny nose. Every question is rated on a 4-point scale, with responses ranging from “0” (no symptoms) to “3” (severe symptoms) [2, 18–20]. The rate of change in TNSS is determined by dividing the absolute difference between TNSS before and after treatment by the TNSS before treatment. When the TNSS rate of change is 0.55 or higher, INCS therapy is effective; if it is below 0.55, it indicates INSC insensitivity [17, 21, 22].

### 2.4. Variables

Duration of AR: The length of the patient's AR is recorded as the interval between the onset of symptoms or diagnosis of AR and their present consultation [10]. AR duration is categorized into the short-term (≤1 year), medium-term (>1 year but ≤3 years), long-term (>3 years but ≤5 years), or ultra-long-term (>5 years).

Age: Patients are classified as young (18–44 years old), middle-aged (45–59 years old), or elderly (older than 60) according to their age bracket.

Marital status: married, single, or others.

Place of residence: Patients are classified as residing in a rural or urban area.

The presence or absence of AR sufferers in the patient's immediate family determines if the patient has a positive or negative family history of AR.

AR can be divided into two subtypes: intermittent AR, which involves rhinitis symptoms that last for less than four days per week or four consecutive weeks, and persistent AR, which features rhinitis symptoms that last for more than four days per week and four consecutive weeks [23].

INCS use can be classified into two types: first-time users and those who have already experienced using INCS.

Comorbidities related to AR can include asthma or others (such as allergic conjunctivitis, atopic dermatitis, sleep-disordered breathing, rhino-sinusitis, and otitis media) [18, 23].

### 2.5. Statistical Analysis

Statistical analysis was conducted using the R 4.2.0 software package (https://www.R-project.org) for this study. A two-tailed *P* value of less than 0.05 was established as the level of statistical significance.

The sample was randomly allocated into training and validation sets in a 7 : 3 ratio. In addition to numerical and proportional representations of categorical variables, Chi-square tests or Fisher's exact tests were employed to evaluate them. Least absolute shrinkage and selection operator (LASSO) regression were applied to select the risk factors. Multivariate logistic regression was used to identify risk factors for INCS insensitivity by examining the associated risk variables. A nomogram was generated using the “rms” package to facilitate the prediction of INCS insensitivity. Calibration plots and Hosmer–Lemeshow (HL) goodness-of-fit tests were used to assess the calibration of the nomogram. Receiver operating characteristic (ROC) curves and areas under the curves (AUC) were calculated for both the training and validation sets to evaluate the accuracy and performance of the nomogram. The clinical utility and net benefit of the predictive models were evaluated using decision curve analysis (DCA).

## 3. Result

### 3.1. Demographic Characteristics of Participants at Baseline

Among the 313 individuals diagnosed with AR, 38.3% were found to be insensitive to INCS therapy. This patient group comprised 64.9% males and 35.1% females. Of these patients, 38.0%, 31.3%, and 30.7% were classified as young, middle-aged, and elderly, respectively. 56.9% of patients had prior exposure to INCS, while the remaining 43.1% were first-time users.  Table 1 shows that no statistically significant differences were observed between the training and validation groups for all nine variables.

### 3.2. Select Predicted Risks

**Figure 2:**
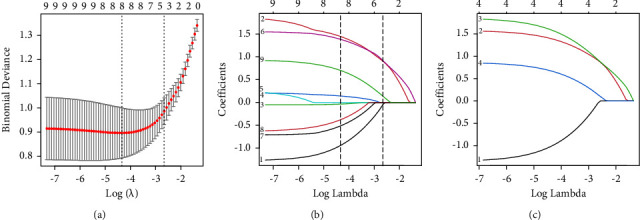
The optimal parameters of the LASSO, with an optimal *λ* of 0.06974 and log (*λ*) of −2.662981, resulting in the reduction of 9 features to 4 (a). (b, c) present the four variables with nonzero coefficients: the type of AR, comorbidities, family history of AR, and the duration of AR.

By employing the LASSO technique, Figure 2 demonstrates the optimal parameter selection. An optimal *λ* of 0.06974 and a log (*λ*) of −2.662981 were chosen, leading to the reduction of nine predictors to four: the type of AR, family history of AR, comorbidities, and duration of AR, as demonstrated in Table 2 after the multivariate logistic regression analysis.

### 3.3. Nomogram of INCS Insensitivity

**Figure 3:**
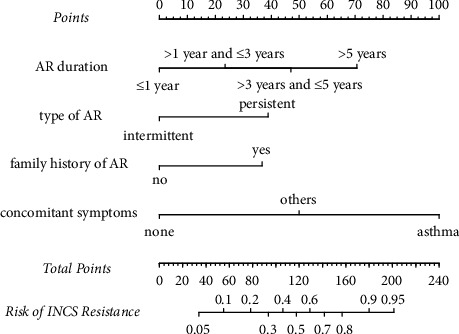
The development of a nomogram to predict an AR patient's probability of INCS insensitivity.

The INCS insensitivity nomogram model was created with LASSO regression screening of four variables (Figure 3). To use the model, draw a vertical line from the “Points” value of each variable on the nomogram and add them down on the “Total Points” axis. This will determine the risk of INCS insensitivity.

### 3.4. Validation of Nomograms

**Figure 4:**
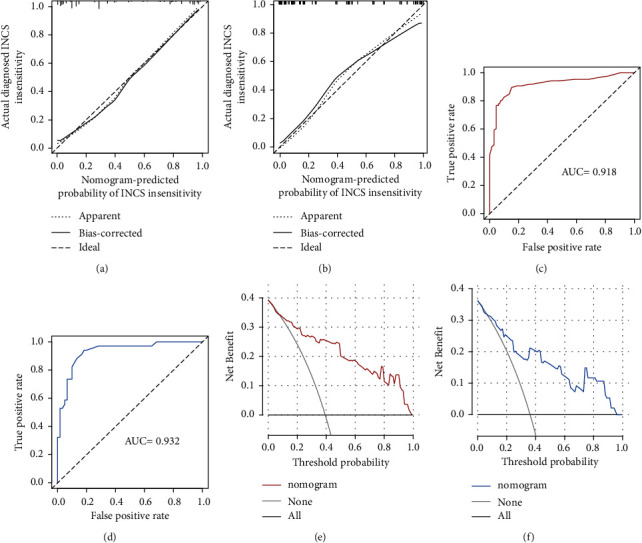
The calibration curves of the nomogram for predicting the risk of INCS insensitivity in the training (a) and validation (b) cohorts. Receiver operating characteristic (ROC) curves were used to assess the model's performance in the training (c) and validation (d) cohorts. Decision curves of the nomogram predicting the risk of INCS insensitivity in the training (e) and validation (f) cohorts are illustrated.

An analysis of Hosmer–Lemeshow (HL) goodness-of-fit determined that the model's performance was satisfactory, as evidenced by a chi-square value of 18.211 (*P* = 0.05) in the training cohorts and 2.988 (*P* = 0.935) in the validation cohorts. In both the training and validation cohorts, the calibration plots of the nomogram demonstrated good agreement between the predicted and observed outcomes. The AUC of the nomogram was measured to be 0.918 (95% confidence interval, 0.859–0.943) in the training dataset and 0.932 (95% confidence interval, 0.849–0.953) in the validation dataset, demonstrating the model's excellent performance. Furthermore, DCA showed that the predictive model had considerable net benefits for the majority of threshold probabilities at various periods of time in the training and validation cohorts, as shown in Figure 4, indicating the potential clinical utility of the predictive model.

## 4. Discussion

Our study included 313 participants with more males than females, and the majority of them ranging in age from 18 to 59 (young to middle-aged adults). This composition was similar to a multi-center, large-scale study of people with AR in China [24], which reported 55.6% of their patients as male and 44.4% as female, with the majority ranging in age from 16 to 55. Consequently, our sample can be seen as representative.

According to a Chinese study [24], 23.2% of patients experienced a worsening of symptoms, and 25.9% saw no change in their condition following treatment. However, there has not been any epidemiological investigation into INCS insensitivity. Since INCS insensitivity can lead to considerable medical and economic costs, it is essential to identify risk factors to ensure the effective use of the drug. Through Lasso and multivariate logistic regression analyses, this study shows that types of AR, accompanying symptoms, family history of AR, and AR duration are all risk factors for INCS insensitivity. These risk factors are simple to recognize, requiring only easily obtained indicators and no invasive procedures such as blood tests. Additionally, a nomogram developed from the aforementioned risk factors displayed high specificity, accuracy, predictive accuracy, and clinical net benefit.

RA patients may be insensitive to INCS due to similar mechanisms as those of fat-soluble glucocorticoid (GC) insensitivity [13], including mutations, reduced or increased expression of glucocorticoid receptor (GR) *α* (GR-*α*) or GR-*α* competitive receptor (GR-*β*), inadequate interaction between GC and GR or between the GR complex and DNA, reduced GR-*α* transport, decreased histone deacetylase expression, and/or enhanced activity of pro-inflammatory transcription factors, although there may be some differences.

A study focused on the GLCCI1 genotype, rs37973 has demonstrated that AR may be insensitive to glucocorticoids due to its inherited nature [25]. This genotype has been associated with asthma patients being less sensitive to inhaled corticosteroids (ICS) [26] and AR patients being less susceptible to INCS [22, 27].

The prolonged and persistent AR might be associated with an increased insensitivity to INCS. This insensitivity can be attributed to the increase in the exposure of allergens in the environment, which leads to a decrease in the expression of GR-*α* and an increase in GR-*β* expression, reducing the body's responsiveness to INCS [28]. Moreover, a study conducted by Fakhri et al. [29] found that ragweed may raise GR expression in the lower turbinates of those affected by AR, along with the upregulation of IL-2 and IL-4 mRNA, making it harder for those suffering from AR to respond to INCS treatment.

It has been observed that the nasal cavity and bronchi are part of the same respiratory tract and that allergies such as AR and asthma share similar pathophysiology [2, 20]. Consequently, it is hypothesized that sufferers of AR and asthma may have impaired GR-*α* transport, as studies have shown that [30], unlike those with hormone-sensitive asthma, the GR-*α* in the cells of the respiratory mucosa cannot be translocated to the nucleus, resulting in glucocortical insensitivity.

Our study was subject to certain limitations, such as selection bias from participants who decided to discontinue the treatment. We found that approximately 38.3% of the participants were insensitive to INCS. However, it is possible that the number of people insensitive to INCS is higher, as those who are insensitive may not continue taking INCS and therefore were not included in our study. Additionally, due to the limited sample size from one site, more data are necessary to validate the accuracy of the constructed nomogram. Despite the drawbacks, this study presents an interesting and unique, more personalized approach for predicting INCS-insensitive AR. It provides healthcare professionals with a new strategy for treating individuals with AR who are unresponsive to INCS.

## 5. Conclusion

This study successfully designed and validated a nomogram for predicting INCS insensitivity in adult patients with allergic rhinitis. The nomogram incorporates important risk factors such as type of AR, comorbidities, family history of AR, and duration of AR. The strong performance of the nomogram on both the training and validation sets indicates its potential as a useful clinical tool for identifying patients who may not respond well to INCS treatment. The decision curve analysis also demonstrated that the nomogram can yield a net clinical benefit for AR patients. This nomogram has the potential to improve personalized treatment and management strategies for Chinese adult patients with allergic rhinitis.

## Figures and Tables

**Table 1:** Summary descriptive table by groups of “INCS insensitivity.”

	All	INCS insensitivity		*P*
	*n* = 313	No (*n* = 193)	Yes (*n* = 120)	
Type of AR				0.029
Persistent	131 (41.9%)	71 (36.8%)	60 (50.0%)	
Intermittent	182 (58.1%)	122 (63.2%)	60 (50.0%)	
Family history of AR:				<0.001
No	166 (53.0%)	134 (69.4%)	32 (26.7%)	
Yes	147 (47.0%)	59 (30.6%)	88 (73.3%)	
Gender				<0.001
Male	203 (64.9%)	102 (52.8%)	101 (84.2%)	
Female	110 (35.1%)	91 (47.2%)	19 (15.8%)	
Age				<0.001
Young	119 (38.0%)	91 (47.2%)	28 (23.3%)	
Mid-aged	98 (31.3%)	58 (30.1%)	40 (33.3%)	
Elderly	96 (30.7%)	44 (22.8%)	52 (43.3%)	
Place of residence				0.016
Rural	162 (51.8%)	89 (46.1%)	73 (60.8%)	
Urban	151 (48.2%)	104 (53.9%)	47 (39.2%)	
Comorbidities				<0.001
None	156 (49.8%)	138 (71.5%)	18 (15.0%)	
Asthma	91 (29.1%)	31 (16.1%)	60 (50.0%)	
Others	66 (21.1%)	24 (12.4%)	42 (35.0%)	
Marital status				<0.001
Married	234 (74.8%)	125 (64.8%)	109 (90.8%)	
Single or others	79 (25.2%)	68 (35.2%)	11 (9.17%)	
First-time users for INCS				0.318
No	178 (56.9%)	105 (54.4%)	73 (60.8%)	
Yes	135 (43.1%)	88 (45.6%)	47 (39.2%)	
Duration of AR				<0.001
≤1 year	108 (34.5%)	91 (47.2%)	17 (14.2%)	
>1 year and ≤3 years	90 (28.8%)	42 (21.8%)	48 (40.0%)	
>3 years and ≤5 years	85 (27.2%)	35 (18.1%)	50 (41.7%)	
>5 years	30 (9.58%)	25 (13.0%)	5 (4.17%)	

**Table 2:** Multivariate logistic regression analysis.

	*β*	Odds ratio (95% confidence interval)	*P*
Type of AR:			
Persistent	Reference	Reference	Reference
Intermittent	−1.336	0.263(0.096∼0.672)	0.007
Family history of AR:			
No	Reference	Reference	Reference
Yes	1.605	4.98(1.138∼25.24)	0.04
Comorbidities:			
None	Reference	Reference	Reference
Allergic conjunctivitis or others	3.608	36.894(9.951∼168.892)	<0.001
Asthma	3.802	44.776(10.462∼241.11)	<0.001
Duration of AR			
≤1 year	Reference	Reference	Reference
1–3 years	17.086	11.51(3.793∼39.118)	<0.001
3–5 years	25.959	32.721(9.306∼139.222)	<0.001
>5 years	4.076	7.29(0.995∼49.645)	0.043

## Data Availability

The data for this study can be obtained upon reasonable request to the corresponding author.
